# Optimized detection of insertions/deletions (INDELs) in whole-exome sequencing data

**DOI:** 10.1371/journal.pone.0182272

**Published:** 2017-08-09

**Authors:** Bo-Young Kim, Jung Hoon Park, Hye-Yeong Jo, Soo Kyung Koo, Mi-Hyun Park

**Affiliations:** 1 Division of Intractable Diseases, Center for Biomedical Sciences, Korea National Institute of Health, Chungcheongbuk-do, South Korea; 2 Macrogen Inc., Gasan-dong, Seoul, South Korea; German Cancer Research Center (DKFZ), GERMANY

## Abstract

Insertion and deletion (INDEL) mutations, the most common type of structural variance, are associated with several human diseases. The detection of INDELs through next-generation sequencing (NGS) is becoming more common due to the decrease in costs, the increase in efficiency, and sensitivity improvements demonstrated by the various sequencing platforms and analytical tools. However, there are still many errors associated with INDEL variant calling, and distinguishing INDELs from errors in NGS remains challenging. To evaluate INDEL calling from whole-exome sequencing (WES) data, we performed Sanger sequencing for all INDELs called from the several calling algorithm. We compared the performance of the four algorithms (*i*.*e*. GATK, SAMtools, Dindel, and Freebayes) for INDEL detection from the same sample. We examined the sensitivity and PPV of GATK (90.2 and 89.5%, respectively), SAMtools (75.3 and 94.4%, respectively), Dindel (90.1 and 88.6%, respectively), and Freebayes (80.1 and 94.4%, respectively). GATK had the highest sensitivity. Furthermore, we identified INDELs with high PPV (4 algorithms intersection: 98.7%, 3 algorithms intersection: 97.6%, and GATK and SAMtools intersection INDELs: 97.6%). We presented two key sources of difficulties in accurate INDEL detection: 1) the presence of repeat, and 2) heterozygous INDELs. Herein we could suggest the accessible algorithms that selectively reduce error rates and thereby facilitate INDEL detection. Our study may also serve as a basis for understanding the accuracy and completeness of INDEL detection.

## Introduction

Recent advances in next-generation sequencing (NGS) technologies have rapidly altered the research and routine work of human geneticists. Specifically, whole-exome sequencing (WES) has been used to elucidate genetic variants underlying human diseases [1]. WES has proven to be a valuable method for the discovery of the genetic causes of rare and complex diseases due to its moderate costs, the amount of manageable data, and straightforward interpretation of results [2, 3].

Several types of natural genetic variations are present in patient samples, including single-nucleotide polymorphisms (SNPs), short insertions or deletions (INDELs) ranging from 1 base (bp) to 10 kilobases (kb) in length, and larger structural variants ranging from 10 kb to several megabases in length. INDELs is a common and functionally important type of sequence polymorphism [4]. This will provide an important resource for applications in medical sequencing, as INDELs have been implicated in a number of diseases [5].

By applying NGS on a large scale, WES is now possible at an individual level [6]. One of the most important aspects of genetics is to identify genetic variants in individuals [1]. INDELs can cause or contribute to human genetic diseases. For example, cystic fibrosis (CF, MIM #219700), neurofibromatosis (NF1, MIM #162200), Charcot-Marie-Tooth neuropathy type 2A (CMT2A, MIM #118210), glycogen storage disease 2 (GSD2, MIM #23200), Huntington disease (HD, MIM #143100), and Duchenne muscular dystrophy (DMD, MIM #310200) are caused by INDELs in the coding regions of DNA. Therefore, the results of INDEL calling from individual WES can be used to predict the future health of individuals and to develop customized medical treatments [7].

Large number of tools are available for short-read alignment and searching for variants (*e*.*g*. SNVs and INDELs). However, the accurate detection of INDELs is still difficult and remains a critical issue. False-positive (FP) and false-negative (FN) rates are critical, especially for genetic diagnosis and Mendelian disease studies. For the future of personalized medicine and genetic diagnosis, highly accurate variant calling remains one of the most important problems [8].

In this study, we used whole exome data from one human genome and analyzed four INDEL detection algorithms: Genome Analysis Toolkit (GATK), Sequence Alignment/Map tools (SAMtools), Dindel, and Freebayes. Here, we show algorithms for available and commonly used methods that detect INDELs and compared their performances using the actual validation data.

## Materials and methods

### Subject

This study examined whole-exome data available from a previous study [9]. Informed consent was obtained from the participant, and the Institutional Review Board of the Korea National Institutes of Health (NIH) approved this study.

### Whole-exome data analysis

Whole-exome libraries were generated from genomic DNA of one individual using the SeqCap EZ Human Exome Library v2.0 (Roche/NimbleGen, Madison, WI, USA) and sequenced using the Illumina HiSeq2000 system (Illumina, San Diego, CA, USA) with paired end reads of 101 bp according to the manufacturer’s protocols. Raw reads in FASTQ format from WES were aligned to the reference genome hg19 using the Burrows-Wheeler Aligner (BWA; http://bio-bwa.sourceforge.net/). Duplicates were removed with Picard (http://picard.Sourceforget.net).

WES data were analyzed using four INDEL calling algorithms, (1) GATK (http://www.broadinstitute.org/gatk/) [10], (2) SAMtools (http://samtools.sourceforge.net/) [11], (3) Dindel [12], and (4) Freebayes [13], following the guidelines provided in the user manuals. INDELs were called with each algorithm and the variants annotated using the ANNOVAR program (http://www.openbioinformatics.org/annovar/).

### Sanger sequencing analysis

INDELs found using the four algorithms were subsequently validated with Sanger sequencing. The Primer3 program (http://frodo.wi.mit.edu/primer3) was used to design primers for amplification of the INDELs identified via exome sequencing. Amplicons from blood genomic DNA were analyzed via gel electrophoresis and were sequenced using an ABI 3730 genetic analyzer (Applied Biosystems, Forster City, CA, USA) with forward and reverse primers.

### Statistical analysis

Their effects on positive predictive value (PPV) and sensitivity were tested using Pearson’s correlation tests. To assess the performance of the different algorithms, we defined several metrics. We defined a call as a true-positive (TP) when WES called a variant and Sanger sequencing detected a variant. A false-positive (FP) call was considered when WES called a variant but Sanger sequencing revealed a wild-type; PPV was calculated as TP/(TP+FP). We defined a false-negative (FN) when Sanger sequencing detected a variant, but the WES called this locus a reference; the sensitivity was calculated as TP/(TP + FN).

## Results

### Performance of INDEL calling in WES

**Fig 1:**
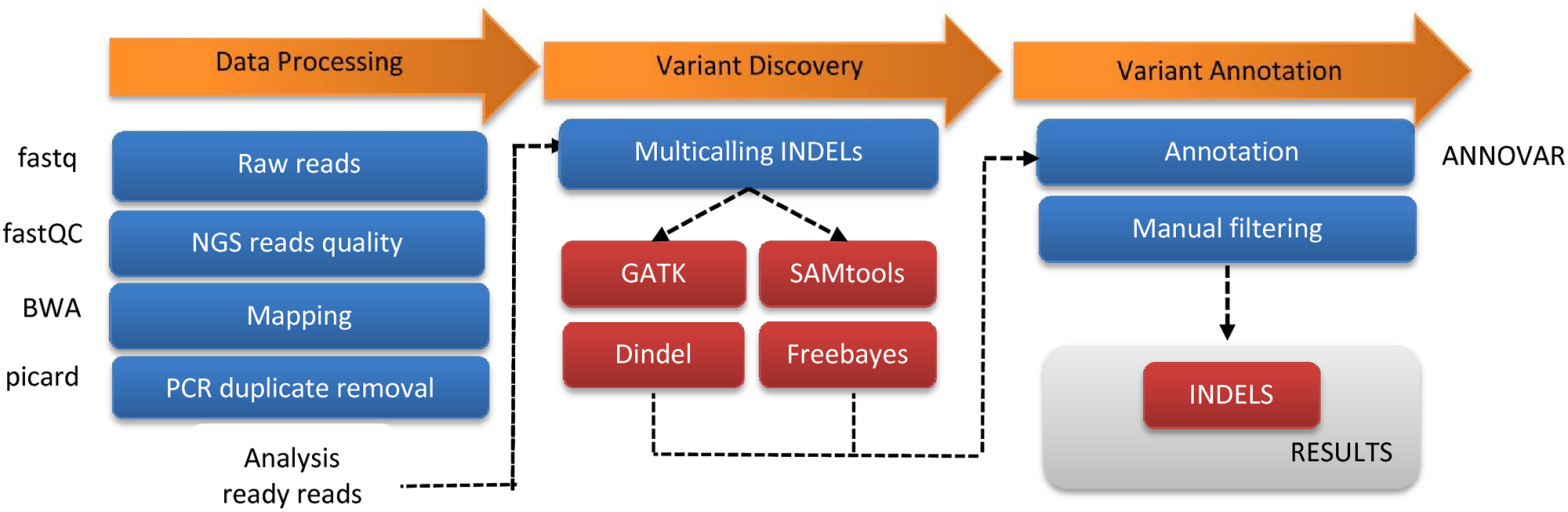
INDEL calling workflow in WES. INDELs were called using four algorithms: GATK, SAMtools, Dindel, and Freebayes. Analysis pipelines and workflow systems are shown.

We provide an analysis pipeline for the detection of INDELs. The genomic pipeline is outlined in Fig 1. For INDEL detection, BAM files were merged so that INDEL calling was performed using four algorithms (*i*.*e*. GATK, SAMtools, Dindel, and Freebayes), and were analyzed. The identified INDELs were then annotated using ANNOVAR to include information such as what gene the variant was in and the consequence of the mutation. S1 Table lists all 840 INDELs identified from the human exome data using the four algorithms.

### Validation of INDELs by Sanger sequencing

**Fig 2:**
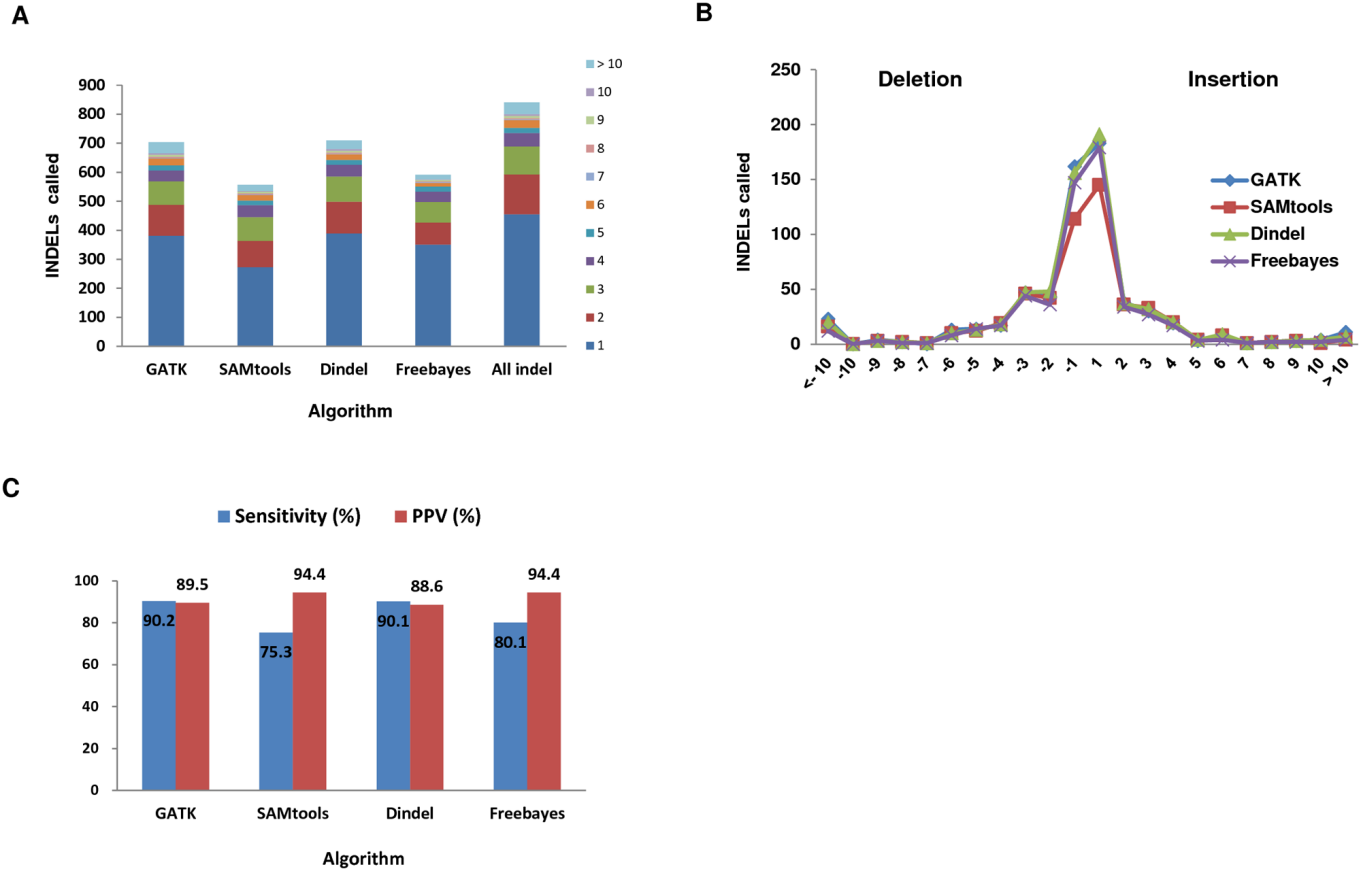
Number of INDELs called by the four algorithms. (A) INDELs were called using four algorithms: GATK, SAMtools, Dindel, and Freebayes. (B) Histograms of insertion (right) and deletion (left) counts by INDEL size. Counts were adjusted within each algorithm to account for the fraction of polarizable calls. (C) Accuracy of detection of INDELs in the four algorithms.

Sanger sequencing was used to evaluate INDEL calling by the four algorithms. The INDEL counts from the four algorithms and validation are presented in Table 1. The 840 INDELs were detected in coding regions and included 429 insertions (51%) and 411 deletions (49%). Fig 2A shows the number of INDELs called by each algorithm. GATK can call INDELs and reported 703 variants and SAMtools identified 556 INDELs. Dindel and Freebayes detected 709 and 591 INDELs, respectively.

**Table 1:** INDELs called and validation in four algorithms.

	INDELs called					Validated					
INDEL size	GATK	SAMtools	Dindel	Freebayes	Total	GATK	SAMtools	Dindel	Freebayes	Total	PPV (%)
**≤ 10**	25	16	20	12	25	23	16	20	12	23	92
**-10**	0	0	1	0	1	0	0	0	0	0	0
**-9**	4	3	3	3	4	4	3	3	3	4	100
**-8**	2	2	2	1	2	2	2	2	1	2	100
**-7**	0	1	1	1	1	0	1	1	1	1	100
**-6**	14	10	10	8	14	13	10	10	8	13	93
**-5**	14	12	13	14	14	14	12	13	14	14	100
**-4**	18	21	20	18	23	17	19	18	17	21	91
**-3**	51	49	53	44	59	47	46	47	44	49	83
**-2**	59	51	68	39	79	43	42	48	36	51	65
**-1**	171	120	174	153	207	162	114	156	147	179	86
**1**	210	152	215	197	248	183	145	191	179	201	81
**2**	47	40	41	37	58	38	36	37	34	45	78
**3**	30	33	34	27	38	30	33	32	27	36	95
**4**	20	20	20	18	23	19	20	20	17	21	91
**5**	3	4	4	3	4	3	4	4	3	4	100
**6**	10	9	10	4	12	8	8	9	4	10	83
**7**	2	1	1	2	2	1	1	1	1	1	50
**8**	2	2	2	2	2	2	2	2	2	2	100
**9**	3	3	3	2	4	3	3	3	2	4	100
**10**	5	2	5	2	5	4	1	4	2	4	80
**> 10**	13	5	9	4	15	11	4	7	4	11	73
**Total**	703	556	709	591	840	629	525	628	558	697	83

We compared the distribution of INDEL sizes called by the four algorithms. All INDEL distributions based on size are shown in Fig 2B. We found that 800 (95%) of the INDELs were 1–10 bp in size. In fact, most INDELs called were ≤ 10 bp, which accounted for 95% (665) of calls by GATK, 96% (535) of calls by SAMtools and Dindel 96% (680), and 97% (575) of calls by Freebayes.

We also examined the overall performance of the four algorithms and computed the sensitivity and positive predictive value (PPV) for each algorithm. The FP and FN number of INDELs called by each algorithm are shown in Table 2. The sensitivity values for GATK, SAMtools, Dindel, and Freebayes were 90.2, 75.3, 90.1, and 80.1%, respectively. The PPVs for GATK (89.5%), SAMtools (94.4%), Dindel (88.6%), and Freebayes (94.4%) were determined by Sanger sequencing (Fig 2C). GATK had the highest sensitivity (90.2%) and SAMtools and Freebayes had the highest PPV (94.4%).

**Table 2:** Validation of the four algorithms used for INDEL calling with WES and Sanger sequencing.

Algorithm	INDELs called	True positive	False positive	False negative	Sensitivity (%)	PPV (%)
**GATK**	703	629	74	68	90.2	89.5
**SAMtools**	556	525	31	172	75.3	94.4
**Dindel**	709	628	81	69	90.1	88.6
**Freebayes**	591	558	33	139	80.1	94.4

PPV: Positive predictive value was calculated by the following: PPV = (#TP/(#TP+#FP))x100, where #TP is the number of true-positive calls, and #FP is the number of false-positive calls.Sensitivity was calculated as follows: (#TP/(#TP+#FN))x100, where #FN is the number of false-negative calls.

### Comparison of INDEL-calling algorithms

**Fig 3:**
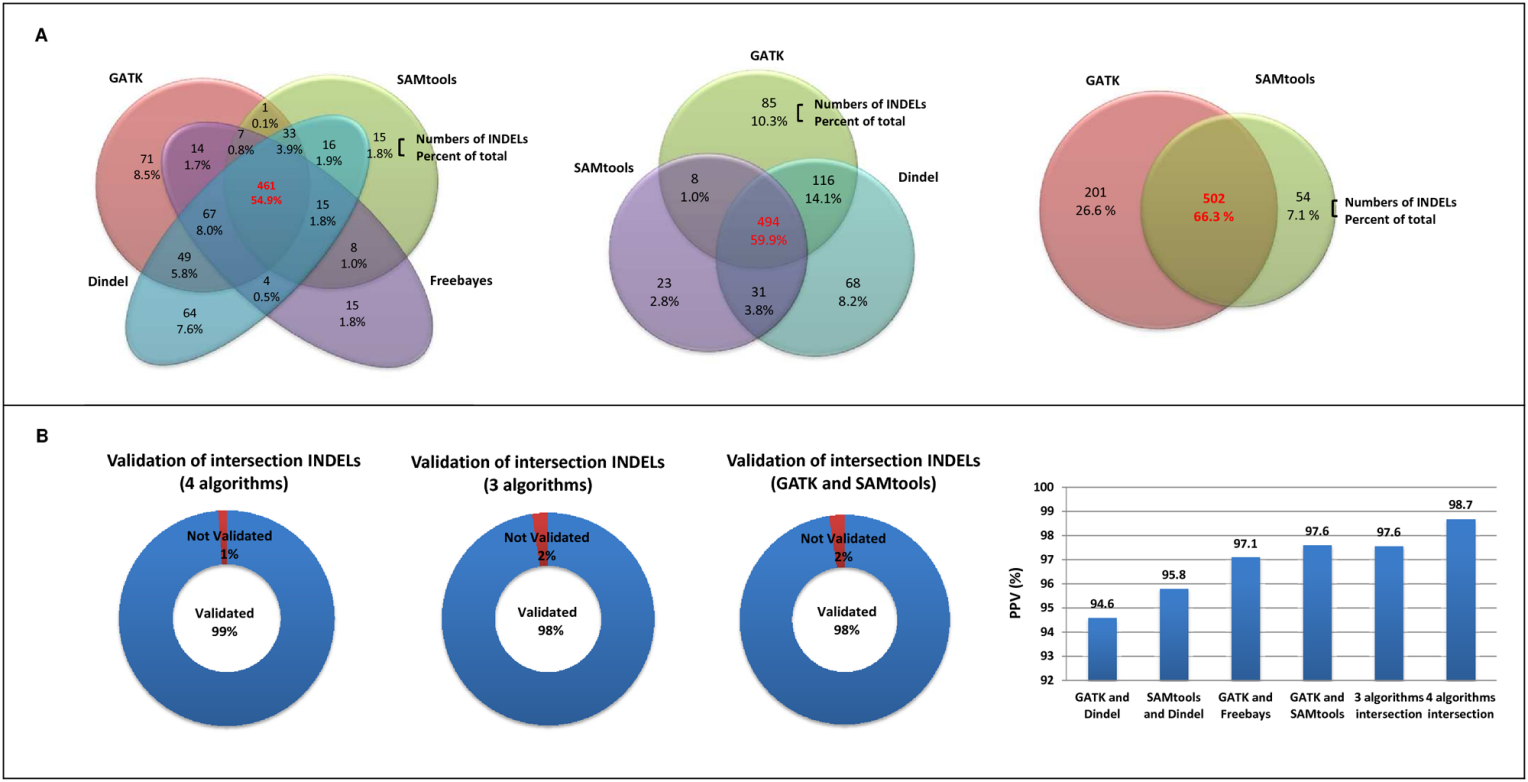
Performance versus detected INDELs and PPVs. (A) Concordance of INDEL detection between the four algorithms: GATK, SAMtools, Dindel, and Freebayes. Venn diagram showing the numbers and percentages of shared INDELs from the four algorithms: 4 algorithm intersection INDELs, 3 algorithm intersection INDELs, 2 algorithm intersection INDELs, and algorithm-specific INDELs. (B) Validation rates and PPVs of the intersecting INDELs between algorithms. The sensitivity increases at higher intersecting algorithms.

We compared the performance of the GATK, SAMtools, Dindel, and Freebayes algorithms for INDEL detection (Table 3). Fig 3 shows the concordance and PPVs of INDELs called by each algorithm and intersection. The concordance for the intersection of the four algorithms (461, 54.9%), three algorithms (494, 59.9%), and GATK and SAMtools (502, 66.3%) were determined (Fig 3A). In addition, the PPV for the four algorithms intersection, the three algorithms intersection, and the GATK and SAMtools intersection INDELs were much higher than those of the intersection for GATK and Dindel, Dindel and SAMtools, and GATK and Freebayes (98.7, 97.6, and 97.6% vs. 94.6, 95.8, and 97.1%, respectively). INDELs were identified with high accuracy (four algorithms intersection: 98.7%, three algorithms intersection: 97.6%, and GATK and SAMtools intersection: 97.6%) (Fig 3B).

**Table 3:** Comparison of INDEL-calling algorithms.

Category	INDELs	True positive	False positive	PPV (%)
**GATK**	703	629	74	89.5
**SAMtools**	556	525	31	94.4
**Dindel**	709	628	81	88.6
**Freebayes**	591	558	33	94.4
**GATK and Dindel**	610	577	33	94.6
**SAMtools and Dindel**	525	503	22	95.8
**GATK and Freebays**	549	533	16	97.1
**GATK and SAMtools**	502	490	12	97.6
**3 algorithms intersection**	494	482	12	97.6
**4 algorithms intersection**	461	455	6	98.7
**heterozygous INDELs**	498	383	115	76.9
**homozygous INDELs**	342	315	27	92.1
**repeat INDELs**	83	29	54	34.9
**non-repeat INDELs**	757	669	88	88.4

**Fig 4:**
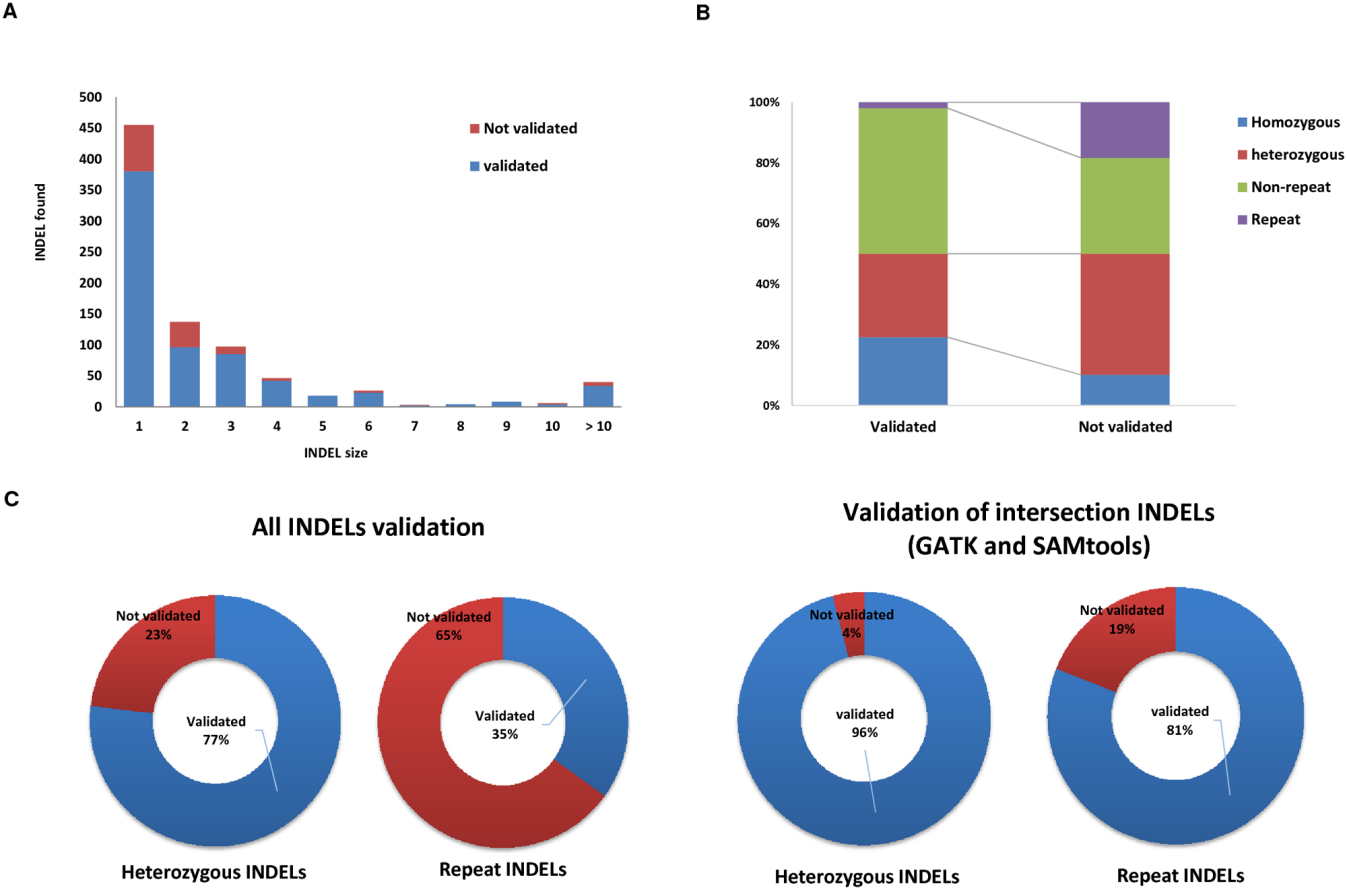
Sources of INDEL detection error from WES. (A) Number of validated INDELs in the following INDEL size. (B) Percentages of homozygous, heterozygous, repeat, and non-repeat in the validated and not validated set. (C) PPVs of error sources, 1) heterozygous, 2) repeat INDELs in all and GATK & SAMtools intersecting call set.

The size distributions of validated INDELs are shown in Fig 4. For the not validated INDELs, there was striking enrichment of heterozygous INDELs (39.9%) and yielded 9.2-fold (2% to 18.4%) more repeat INDELs than validated set. The PPVs of heterozygous INDELs (76.9%), homozygous INDELs (92.1%), repeat INDELs (34.9%), and non-repeat INDELs (88.4%) were also calculated. We found that the validation rate of heterozygous and repeat INDELs for GATK and SAMtools intersection increase with 96.0 and 81.0%.

## Discussion

In this study, we investigated the performance of tools available for the INDEL detection from WES data. We evaluated four publicly available algorithms that are well-known for calling short INDELs. We provide an analysis pipeline for the detection of INDELs so that INDEL calling were performed using four algorithms (*i*.*e*. GATK, SAMtools, Dindel, and Freebayes) to identify TP INDEL calls while reducing FP calls.

Many studies have reported the INDEL calling capabilities of available tools from NGS data [14–18]. Previous evaluation by Neuman *et al*. was based on simulated data [14]. Notably, only random selected 215 INDELs were validated [15, 16]. However, our study used actual validation data. We reported 840 INDELs called from the four programs in one human genome, all of these INDELs were validated by Sanger sequencing.

GATK is a collection of analysis tools for human data that was developed by the Broad Institute. GATK performs variant calling using HaplotyperCaller (HC) [10]. SAMtools is based on a Bayesian model for INDEL calling, which parses SAM and BAM files and includes BCFtools to call SNPs and short INDELs from a single alignment [11]. Dindel is a program developed by the Wellcome Trust Sanger Institute that uses a Bayesian approach for calling INDELs from NGS data [12]. Freebayes is a Bayesian genetic variant detector designed to find SNPs, INDELs, MNPs, and complex events smaller than the length of a short-read sequencing alignment [13].

The GATK’s model is derived from Dindel’s model, where GATK is expected to show similar performance to Dindel. Freebayes is a haplotype-based caller, similar to GATK; however, GATK contains additional algorithms for filtering with low mapping quality and local realignments (http://software.broadinstitute.org/gatk/) [19]. SAMtools may improve the processing of INDELs through likelihood algorithms, such as the indel genotype likelihood model, genotype-free analysis, and physical phasing (http://samtools.sourceforge.net/) [19, 20].

In our actual validation data, a total of 629 true positive INDELs in GATK and 628 in Dindel were identified. GATK and Dindel had the least FNs and the highest number of TPs, showing sensitivity of 90.2% (GATK: 629 of 697) and 90.1% (Dindel: 628 of 697), respectively. We also examined the positive predictive value (PPV) for the two algorithms, and GATK had a higher PPV than Dindel (89.5 vs. 88.6%). On the other hand, SAMtools and Freebayes had the least FPs. By decreasing the false positive rate, the accuracy (PPV) of SAMtools and Freebayes improved to 94.4% (525 of 556) and 94.4% (528 of 591), but it reduce the power of true positive INDEL detection. The GATK and SAMtools intersection INDELs were much higher than those of the intersection for GATK and Dindel, Dindel and SAMtools, and GATK and Freebayes. Based on these results, GATK had the fewest FN calls, while SAMtools had the fewest FP calls. Thus, GATK had high sensitivity, while SAMtools had high accuracy. Collectively, GATK and SAMtools complement the strengths and weaknesses of the other algorithm to yield superior results.

We compared the distribution of INDEL size called by the four algorithms. Most INDELs called by the algorithms were ≤10 bp. The statistical tests showed that the distribution of INDEL size did not differ significantly among the algorithms. In other words, INDEL size is not a confounding factor that affects the performance of these calling algorithms.

To determine the error of INDEL call from WES data, INDELs were compared based on where they were repeats or heterozygous. The PPVs for heterozygous and repeat INDELs were 76.9 and 34.9%, respectively, while homozygous and non-repeat INDELs were validated 92.1 and 88.4%. For the heterozygous and repeat INDELs called by both GATK and SAMtools, 96.0 and 81.0%, were successfully validated.

GATK had the highest sensitivity of all the algorithms, while SAMtools had high PPV. Thus, we recommend that GATK and SAMtools be used in combination for the detection of INDELs. GATK and SAMtools show better performance in calling INDELs than Dindel and Freebayes. Additionally, two key sources of difficulties in accurate INDEL detection are the presence of repeats and heterozygous INDELs. Our study may also serve as a basis for understanding the accuracy and completeness of INDEL detection. We believe that our method is a useful tool for understanding human diseases through WES analysis.

## Supporting information

S1 Table Summary of INDELs called by GATK, SAMtools, Dindel, and Freebayes. (XLSX)
